# Metformin modulates alveolar bone parameters and gene expression in streptozotocin-induced type 2 diabetic rats

**DOI:** 10.1186/s12903-026-10091-1

**Published:** 2026-11-05

**Authors:** Israa Ahmed Radwan, Gamilah Al-Qadhi, Hanaa S. Mansy, Sana Mostafa, Sara El Moshy

**Affiliations:** 1 Oral Biology Department, Faculty of Dentistry, Cairo University, Cairo, Egypt; 2 Stem Cells and Tissue Engineering Research Group, Faculty of Dentistry, Cairo University, Cairo, Egypt; 3 Department of Basic Dental Sciences, Faculty of Dentistry, University of Science and Technology, Aden, Yemen; 4 Department of Oral and Maxillofacial Radiology, Faculty of Dentistry, Cairo University, Cairo, Egypt

**Keywords:** Type 2 diabetes, Streptozotocin, Metformin, Alveolar bone, Bone healing

## Abstract

**Background:**

Diabetes mellitus (DM) is linked with defective bone healing and metabolic disturbances that increase fracture risk. Although metformin (MET) is used traditionally as first-line treatment for type 2 diabetes (T2DM), its effectiveness in completely reversing diabetic bone homeostasis is uncertain. The present study was conducted to assess the short-term effect of MET on alveolar bone histological structure and osteoblastic gene expression in rats with streptozotocin (STZ)-induced type 2 diabetes mellitus (T2DM).

**Methods:**

Eighteen adult male Wistar rats were divided equally into three groups (*n* = 6/group): control group (non-diabetic) was injected with saline, STZ group (diabetic) received 10% fructose solution for 2 weeks followed by an injection of a single dose of STZ (40 mg/kg), and MET group (diabetic with MET-treatment) received 100 mg/kg/day MET for 30 days. Alveolar bone was assessed by histological and histomorphometric analysis, cone beam computed tomography (CBCT), and reverse transcription-polymerase chain reaction (RT-PCR) analysis of bone-related gene expression.

**Results:**

The STZ group showed pronounced decreases in total bone area, osteoblast area, and bone-related gene expression (RunX2, osteocalcin, COL1A1) compared to the control group. MET significantly improved these parameters compared to the STZ group.

**Conclusions:**

Metformin significantly increased total bone area and osteoblastic gene expression (Runx2, osteocalcin, and COL1A1) compared to untreated diabetic rats but did not fully restore them to control (non-diabetic) levels. These results suggest a partial protective effect of metformin in alveolar bone in this short-term T2DM rat model.

## Introduction

Diabetes mellitus (DM) is one of the twenty-first century’s fastest-growing global health challenges. DM is a significant metabolic disorder characterized by persistent hyperglycemia resulting from either a deficiency in insulin secretion, as seen in type 1 diabetes mellitus (T1DM), resistance to insulin action, as seen in type 2 diabetes mellitus (T2DM), or a combination of both [1]. Globally, DM was the direct cause of 1.5 million deaths in 2019 [2]. It is predicted to emerge as the most significant driver of disability and mortality by 2030 [2, 3]. As a result, DM imposes a considerable burden on society and has a substantial economic impact on individual medical costs and healthcare systems [4].

Over 90% of patients with diabetes have T2DM [5]. T2DM is characterized by hyperglycemia, which leads to the generation of reactive oxygen species (ROS) and a redox imbalance, thereby triggering oxidative stress and impairing carbohydrate, lipid, and protein metabolism [1, 6].

The overproduction of ROS modulates several intracellular signaling pathways that lead to insulin resistance (IR), impairment of β-cell function, formation of glycation end products (AGEs), as well as micro- and macrovascular diabetic complications [6]. The development of IR is a critical factor in the etiology and pathogenesis of T2DM, primarily linked to low-grade tissue-specific inflammatory responses triggered by numerous pro-inflammatory mediators [7].

This dysregulation and reduced insulin signaling may adversely affect bone cells, namely osteoblasts and osteoclasts. T2DM is associated with lower bone turnover, which interferes with bone formation, increases the risk of fracture, and impairs fracture healing [8]. The jawbone of patients with T2DM exhibits changes in microstructure, mineralization, and osteocyte shape [9, 10].

Compared to non-diabetic individuals, T2DM patients were reported to have an increased risk of osteoporosis or osteopenia and a higher early-stage failure rate of dental implants [11, 12]. Furthermore, a recent review concluded that the risk of periodontitis and peri-implantitis increased in DM patients, which is directly related to their glycemic control [13].

Metformin (MET) is the first-line treatment for T2DM worldwide. It has gained increasing interest recently for its potential to improve bone healing, decrease the probability of fractures, and improve IR [14]. MET acts through multiple cellular pathways, particularly involving the activation of AMP-activated protein kinase (AMPK), to counteract IR and decrease blood glucose levels. This effect restores mitochondrial function, enhances lipid metabolism, and reduces inflammation [15, 16]. It was documented that early intervention with MET treatment leads to better glycemic control than delayed treatment [17].

The impact of MET in bone metabolism is still controversial, previous research has revealed that MET can enhance glucose metabolism, reduce alveolar bone loss, promote the repair of damaged bone and promote osteoblasts’ proliferation, differentiation, and mineralization [18, 19], Nonetheless, it was also reported that MET had no significant effect on bone mineral density and it even decreased some bone turnover markers [20]. Therefore, further research is needed to expand current knowledge and strengthen the evidence on this topic.

Radiographic assessment is crucial for determining bone quantity and quality during dental implant and surgical procedures [21]. Cone-beam computed tomography (CBCT) is a valuable three-dimensional (3D) imaging modality that provides accurate and precise measurements of bone quantity and can estimate the density of the jawbone [22]. CBCT enables clinicians to evaluate the surgical or implant site in three orthogonal planes, which helps them gain complete 3D data about the assessed area. Most surgical and implant treatment-planning decisions recently rely on information derived from CBCT [23].

In jaw tissue, runt-related transcription factor-2 (Runx2), osteocalcin (OCN), and collagen Type I (COL1A1) are three key genes that regulate the process of bone formation. Runx2 activation and expression provide the initial signal for osteoblast differentiation, triggering the transcription of downstream osteoblast-characteristic genes, including OCN and COL1A1. Osteocalcin is a later-stage marker of mature osteoblasts and is involved in bone mineralization, while COL1A1 encodes the most abundant protein in the bone extracellular matrix [24]. As T2DM has been shown to alter the expression of key bone-forming genes, it could serve as a marker of an osteogenic condition [25].

Animals have been used extensively as models to research the consequences of DM. Streptozotocin (STZ) is a widely used agent for DM induction, as it selectively destroys pancreatic β-cells in the islets of Langerhans, leading to insulin insufficiency and hyperglycemia [26].

The effectiveness of antidiabetic medications on the alveolar bone dynamics at the early onset of disease is yet to be investigated. The implications of the short-term effect of MET on bone metabolism and bone cells in T2DM compared to the non-diabetic population are still controversial. Therefore, the current study aimed to evaluate the effects of MET on alveolar bone parameters and osteoblastic gene expression in rats with STZ-induced T2DM, utilizing histological, histometric, molecular, and radiographic analyses. Based on this aim, we hypothesize that MET treatment will significantly improve alveolar bone morphology, osteoblastic gene expression, and bone histomorphometric parameters in STZ-induced T2DM rats compared to untreated diabetic controls.

## Methods

### Ethical approval

The present study was approved by the Institutional Animal Care and Use Committee (IACUC) at Cairo University (Approval code: CU III F C 53 23). It was conducted in compliance with the ARRIVE guidelines and regulations (https://arriveguidelines.org). No anesthesia was administered to the animals during the study procedures. The degree of pain associated with intraperitoneal injection and oral gavage was considered minimal, momentary, or transient and did not necessitate the use of anesthetic agents; this approach was reviewed and approved by the IACUC in accordance with the ARRIVE guidelines.

### Sample size calculation

Considering the bone volume outcome from a previous study [27], a total sample of 18 rats (6 per group) was deemed sufficient to detect an effect size of 1.88, with 80% power, a two-sided hypothesis test, and a significance level of 0.05. The sample size was calculated using the G*power program [28], based on an independent samples T-test.

### Experimental animals

Eighteen adult male Wistar rats, weighing 220–250 g (8–10 weeks of age), were obtained from and housed in the animal house of the Faculty of Medicine, Cairo University. The animals were bred and supplied by this institutional animal facility and were not privately owned by any individual, farm, or external institution. The rats were maintained in a controlled environment with a room temperature of 22 ± 3 °C and a humidity level of 50–60%, along with the required 12-hour light-dark cycle. Animals were housed under standard laboratory conditions with free access to food and water.

### Study design

After a one-week acclimatization period, the rats were randomly distributed into three groups (one negative control and two experimental), each including six animals, as follows: *control group*: rats were not exposed to any treatment and were allowed a regular diet with a single IP saline injection. *STZ group* (non-treated diabetic group): T2DM was induced by a single IP injection of STZ at 40 mg/kg body weight, without treatment [29]. *MET group*: (MET-treated diabetic group) T2DM was induced as in the previous group, followed by daily oral administration of MET 100 mg/kg body weight [30]. Doses of MET were administered by oral gavage using a needle fitted to a disposable syringe.

### Induction of T2DM by STZ

Before the start of the experiments, the blood glucose levels of each rat were measured using a Fine test™ Auto Coding Premium blood glucose meter (Osang Healthcare Co., Ltd, South Korea). The blood from each animal was collected from the tail vein. The blood glucose levels for rats ranged from 75 to 115 mg/dl.

**Fig. 1:**
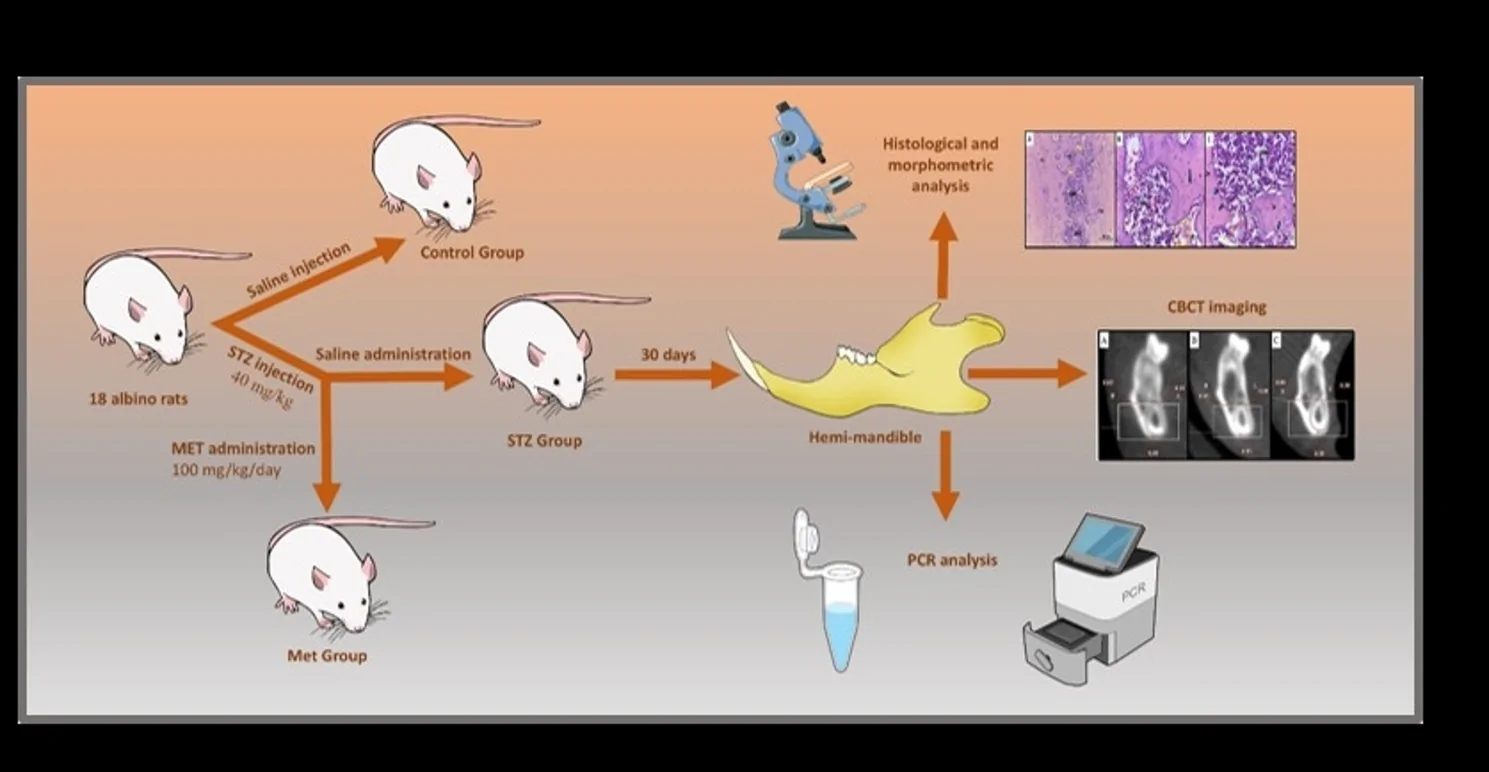
Schematic representation of the experimental timeline, including induction of T2DM by 10% fructose feeding followed by a single intraperitoneal injection of streptozotocin (STZ, 40 mg/kg), and the 30-day metformin (MET, 100 mg/kg/day) treatment period in the three study groups (control, STZ, and MET)

T2DM was induced in groups 2 and 3 according to previous work [29], where drinking water was replaced with a 10% fructose solution for a couple of weeks to induce insulin resistance. A dose of 40 mg/kg body weight of STZ (Sigma, St. Louis, MO, USA) was dissolved in freshly prepared 0.01 M citrate buffer (pH 4.5) and injected intraperitoneally in rats of the experimental groups to induce partial pancreatic β-cell dysfunction. In contrast, the control group received an injection of comparable saline. Three days after induction of T2DM, rats with non-fasting blood glucose levels higher than 300 mg/dl were considered diabetic and randomly distributed among the two experimental groups. The blood glucose levels and weights were assessed once per week till the day of euthanasia. All animals were checked daily for any behavioral changes and abnormal clinical signs. This combined fructose-feeding and low-dose STZ protocol recapitulates the dual pathophysiology of human T2DMand is therefore distinguished from high-dose STZ models, which produce the absolute insulin deficiency characteristic of T1DM (Fig. 1)**.**

### Tissue preparation

Before sacrifice, weights and blood glucose levels were measured. Animals were euthanized 30 days after confirmation of T2DM induction and group allocation, corresponding to completion of the 30-day MET treatment period with an intravenous overdose of sodium thiopental (80 mg/kg) (Sigma Tec Pharma – Egypt) [31], and death was confirmed by head decapitation. The jaws of all rats were separated into hemi-mandibles and prepared for histological and histomorphometric evaluation and molecular analysis using reverse transcription polymerase chain reaction (RT-PCR), using six samples (hemi-mandibles) per group for each analysis. CBCT imaging was performed on a representative subset of samples per group.

### Histopathological examination

The samples were immediately immersed in 10% neutral formalin for 24–72 h, after which they were thoroughly rinsed to remove any residual fixative. For decalcification, the tissues were immersed in a 10% solution of ethylenediamine-tetraacetic acid (EDTA) [32]. Following a four-week duration, the samples underwent dehydration using progressively higher concentrations of ethyl alcohol, were subsequently cleared in xylene, and were then embedded in paraffin. Sections measuring 4–5 μm were stained with hematoxylin and eosin (H&E) and subsequently analyzed using a Leica RM2255 light microscope equipped with a digital camera and image analysis software.

### Histomorphometric analysis

**Fig. 2:**
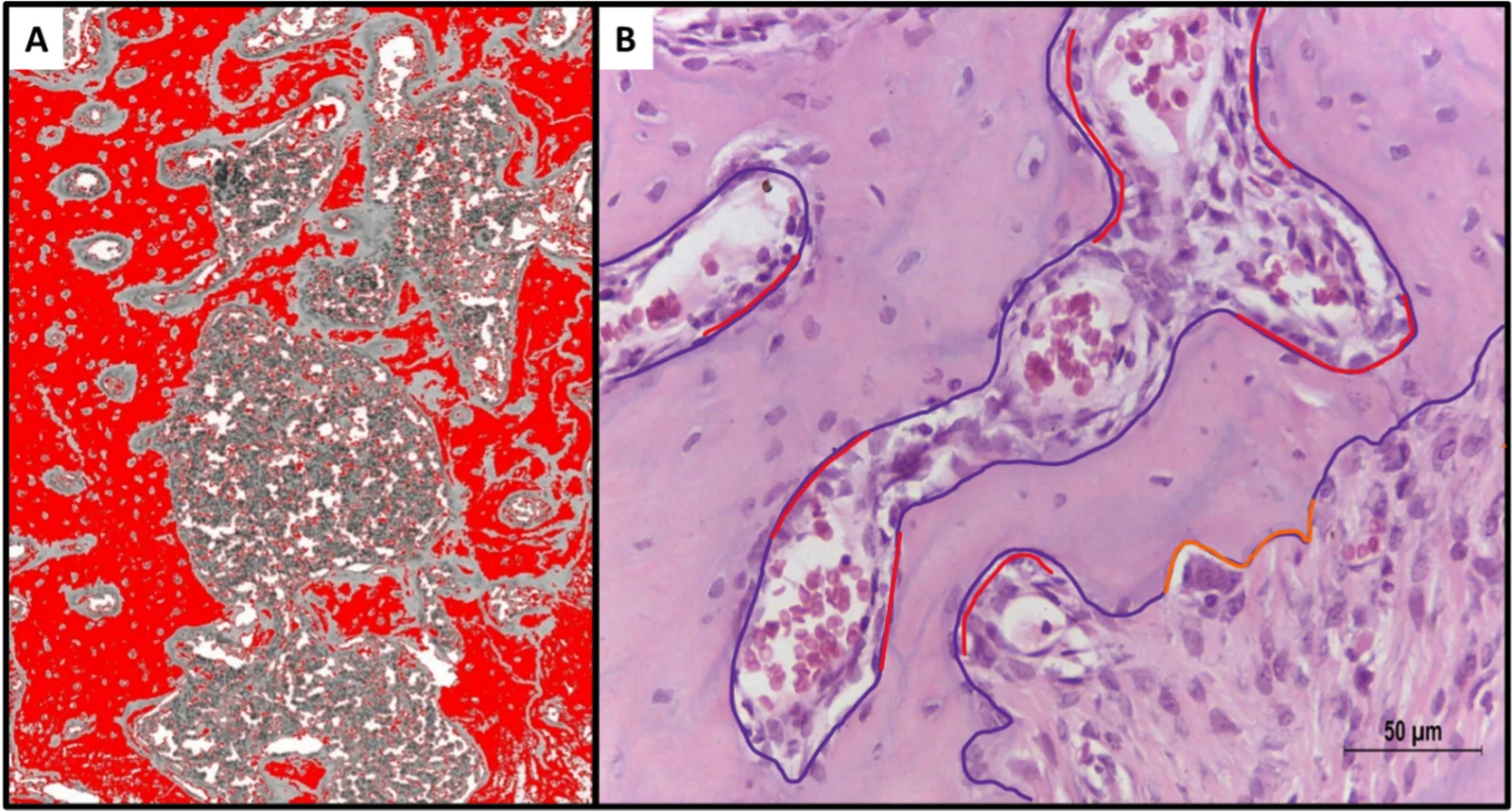
**A** Total bone area percentage masked with binary colour as measured via ImageJ software. **B** Osteoblast area/bone area and resorbing area/bone area measured via ImageJ software. The red line represents the perimeter of bone covered by osteoblasts, the orange line represents the perimeter of bone covered by eroded cavities. In contrast, the blue lines represent the total bone perimeter

In H&E-stained sections, Fiji ImageJ (image analysis software) [33] was used to analyze the percentage of bone area, as well as osteoblast area/bone area and resorbing area/bone area, as previously described [34–36]. Bone area percentage was measured on images magnified at X10; it can be defined as the percentage of calcified bone in relation to the total image area **(**Fig. 2-a**).** Osteoblast area/bone area is defined as the ratio of the bone perimeter occupied by osteoblasts as compared to the total bone perimeter, while resorbing area/bone area is defined as the ratio of bone perimeter occupied by eroded cavities to total bone perimeter **(**Fig. 2-b**).** The osteoblast area/bone area and resorbing area/bone area were measured on images magnified at X40. Image analysis was performed on images acquired with X10 and X40 magnification. A total of 10 fields were measured, and the mean values were calculated for each sample. All measurements were acquired from the interradicular septum between the roots of the mandibular first molars. One calibrated, blinded examiner performed all measurements.

### Reverse transcription polymerase chain reaction (RT-PCR)

Samples were immediately frozen and stored at − 80 °C until RNA extraction. RT-PCR was conducted to quantify the relative expression of runt-related transcription factor-2 (RunX2), osteocalcin, and collagen Type I (Col1A1) genes in the inter-radicular septum between the roots of the mandibular first molars. Following the manufacturer’s guidelines, total RNA was extracted with the QIAzol lysis reagent (Qiagen, Venlo, the Netherlands). The amount and integrity of RNA were assessed using a Nanodrop ND-1000 spectrophotometer (Nanodrop Technologies).

For quantitative assessment of RunX2, osteocalcin, and Col1A1 mRNA levels, 5 µL of total RNA from each sample was used to produce complementary DNA (cDNA) by reverse transcription. cDNA was generated via a cDNA synthesis kit (Applied Biosystems, Waltham, USA). The cDNA was amplified using the BioRad SYBR^®^ Green qPCR Master Kit. RT-PCR amplification was performed using a StepOnePlus™ (Applied Biosystems) system under standard cycling conditions. The GAPDH gene served as the internal control, and gene expression was normalized against the mean critical threshold (CT) values with the ΔΔCT method. Within the current study, GAPDH was used as a house keeping gene, similar to a recent previous study on T2D [37], as it shows an abundant and stable expression in various rodent tissues [38–42] and it remains relatively stable in T2D [43, 44]. The data was analyzed via Rotor-Gene 6000 Series Software, version 1.7. The primer sequences specific to each gene are illustrated in Table 1.

**Table 1:** Primer sequence of the analyzed genes

Gene	Primer sequence
RunX2	**F** 5’-CCTGAACTCTGCACCAAGTCCT-3’. **R** 5’-TCATCTGGCTCAGATAGGAGGG − 3’.
Osteocalcin	**F** 5’- GCAATAAGGTAGTGAACAGACTCC-3’. **R** 5’- GTTTGTAGGCGGTCTTCAAGC − 3’.
Col1A1	**F** 5’-GATTCCCTGGACCTAAAGGTGC − 3’. **R** 5’- AGCCTCTCCATCTTTGCCAGCA-3’.
GAPDH	**F** 5’-CATCACTGCCACCCAGAAGACTG − 3’. **R** 5’-ATGCCAGTGAGCTTCCCGTTCAG − 3’

### Radiographic assessment

A CBCT scan was performed on a selection of three representative hemi-mandibles from each group using a Planmeca ProMax^®^ 3D Mid CBCT machine (Helsinki, Finland) at the Oral and Maxillofacial Department, Faculty of Dentistry, Cairo University. Each hemi-mandible was scanned using exposure parameters of 90 kVp, 12 mA, and 15 s, with a voxel size of 75 μm, following the exposure parameters used by Kim et al. (2012) [45] but with a smaller voxel size. The radiographic assessment and data extraction were executed by an Oral and Maxillofacial Radiologist with 15 years of experience who was completely blinded to the experimental groupings. The lower first molar furcation area was assessed to evaluate the following: the width of the bone marrow spaces subjectively, the cortical bone thickness of the inferior border of the mandible, buccal and lingual cortices, in addition to the average gray value in the area above the mandibular canal and below the furcation area of the mandibular first molar.To radiographically assess the healing progression, the Average Gray Value (AGV) was computed in the predetermined regions of interest. In the present study, the AGV was used as an indirect radiographic marker of X-ray beam attenuation in the region of interest, reflecting the extent of bone filling and marrow space occupancy, rather than a direct measure of bone mineralization or tissue composition. As expected, with more radiographic bone filling of the marrow spaces, higher AGV values are associated with more attenuation of the X-ray beam [46].

### Subjective evaluation of the marrow spaces

An explorer module showing the axial, coronal, and sagittal CBCT images was selected. The corrected coronal cut was adjusted at the furcation area for the lower first molar. Serial corrected coronal cuts were assessed to evaluate the marrow spaces, comparing the groups to each other.

### Measurements of cortical thickness

On the central corrected coronal cuts, the measurements of the cortical bone thickness were done. For standardization of the measurements, a box tool was selected to draw a box between the superior cortical border of the mandibular canal and the inferior border of the mandible. The superior border of the box was tangent to the superior border of the mandibular canal, and the inferior border of the box was tangent to the inferior border of the mandible. The buccal and lingual cortical thickness was measured at the superior border of the drawn box. In contrast, the thickness of the inferior border of the mandible was measured perpendicular to the inferior border of the drawn box.

### Estimated average gray value assessment

On the corrected coronal cut, a box was drawn in the area below the furcation area and above the mandibular canal for measuring the estimated average gray value of this area, following the methodology of Cassetta et al. (2014) [47]. The AGV indicates X-ray beam attenuation within the selected region of interest, with higher values representing greater attenuation.

### Statistical analysis

Data from blood glucose levels, body weight, PCR analysis (Runx-2, Osteocalcin, and Collagen Type I), in addition to data from histomorphometric analysis (bone area percentage, osteoblasts area/bone area, and resorbing area/bone area), was checked for normality using Shapiro-Wilk test and homogeneity using Levene’s test. All data sets (blood glucose levels, body weight, Runx-2, Osteocalcin, Collagen Type I, bone area percentage, osteoblasts area/bone area, and resorbing area/bone area) were normally distributed. Data sets were presented as mean and standard deviation (SD). Student’s T test was used for comparing 2 groups, comparisons between more than 2 groups was performed using one-way analysis of variance (ANOVA) test, followed by Tukey’s post hoc test when the ANOVA showed significant results, while data regarding blood glucose levels and body weight were analyzed using a two-way mixed effects ANOVA with time as the repeated measure, followed by Tukey’s post hoc test. The significance level was set at *p* < 0.05. Statistical analysis was performed using SPSS 27 (Statistical Package for the Social Sciences, SPSS, Inc., Chicago, IL, USA) for Windows.

## Results

### Blood glucose levels and animal’s body weight

**Fig. 3:**
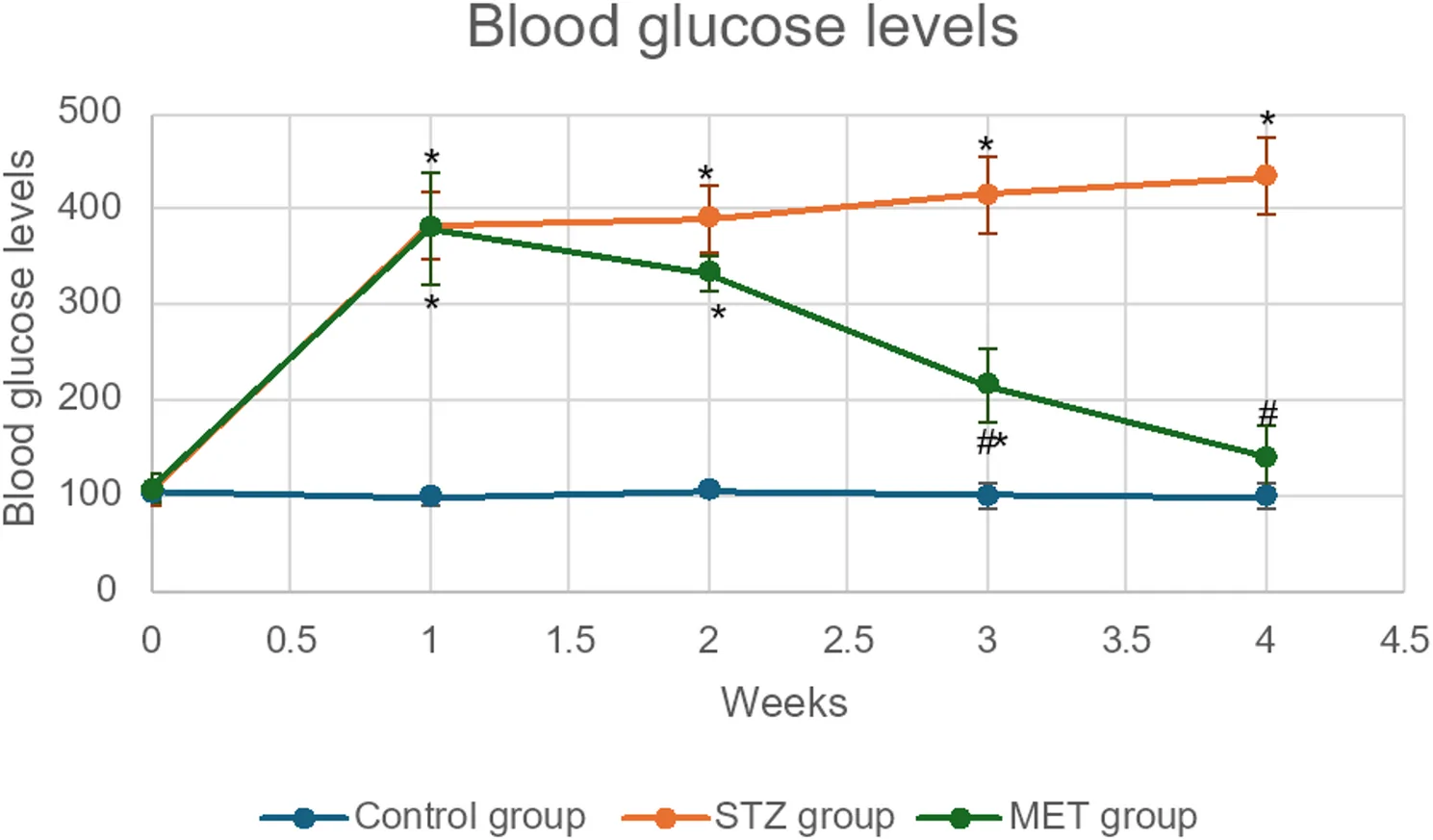
Mean blood glucose levels over the experiment duration with SD error bars. *; *P* < 0.05 vs. control group, #: *P* < 0.05 vs. MET group

A significant increase in blood sugar levels was observed in STZ group as compared to control group in weeks 1, 2, 3, and 4, and in MET group as compared to control in weeks 1, 2, and 3. A significant reduction in blood sugar levels was observed in MET group as compared to STZ group in weeks 3, and 4. **(**Table 2; Fig. 3**).**

While STZ group showed a significant increase in blood glucose levels at week 4 as compared to week 1, MET group showed an insignificant difference in blood glucose levels at week 4 as compared to week 1. **(**Table 2; Fig. 3**).**

**Fig. 4:**
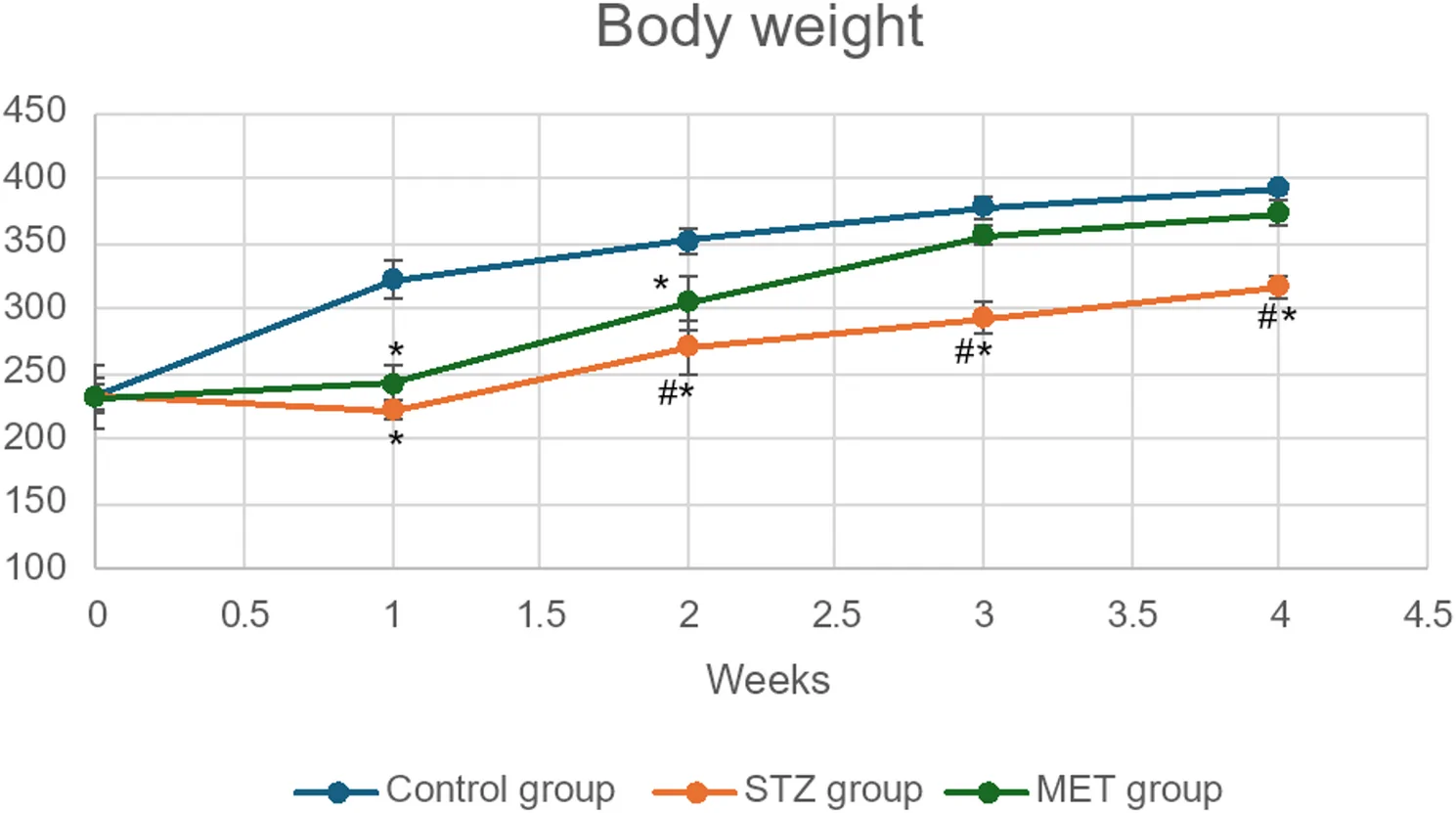
Mean body weight over the experiment duration with SD error bars. *; *P* < 0.05 vs. control group, #: *P* < 0.05 vs. MET group

A significant decrease in body weight was observed in STZ group compared to control group in weeks 1, 2, 3, 4, also in MET group compared to control in weeks 1 and 2. A significant increase in body weight was observed in MET group as compared to STZ group in weeks 2, 3, and 4. Both STZ and MET groups showed a significant increase in body weight at week 4 as compared to week 1. **(**Table 3; Fig. 4**).**

**Table 2:** Descriptive statistics and comparison between blood glucose level in all studied groups (Two-way mixed effects ANOVA and Tukey’s post hoc test)

Blood glucose level	Week 0	Week 1	Week 2	Week 3	Week 4	*P* value
Control	103.8 ± 7.73 ^D^	98.8 ± 7.94 ^D^	106.5 ± 5.21 ^D^	101.17 ± 13.70 ^D^	99.8 ± 13.36 ^D^	*P* < 0.001*
STZ	106 ± 16.42 ^D^	383 ± 40.1 ^A, B^	390 ± 40.3 ^A, B^	416.3 ± 35.3 ^A^	434.2 ± 33.8 ^A^	
MET	106.8 ± 15.28 ^D^	380.7 ± 59.1 ^A, B^	333 ± 19.20 ^B^	216.2 ± 38.7 ^C^	140.3 ± 33.7 ^D^	

Significance level *P* < 0.05, *significant

Means with different superscript letters are significantly different

**Table 3:** Descriptive statistics and comparison between body weight level in all studied groups (Two-way mixed effects ANOVA test and Tukey’s post hoc test)

Body weight	Week 0	Week 1	Week 2	Week 3	Week 4	*P* value
Control	232.7 ± 25.0 ^F^	322 ± 14.13 ^C^	352.17 ± 10.11 ^B^	378 ± 8.05 ^A, B^	392.5 ± 3.78 ^A^	*P* < 0.001*
STZ	233 ± 13.28 ^F^	222 ± 7.56 ^F^	270.33 ± 20.19 ^E^	292.5 ± 12.28 ^D, E^	316.5 ± 8.09 ^C, D^	
MET	231.67 ± 9.24 ^F^	242.5 ± 13.28 ^F^	304.33 ± 20.26 ^C, D^	356.67 ± 7.69 ^B^	373.5 ± 9.61 ^A, B^	

Significance level *P* < 0.05, *significant

Means with different superscript letters are significantly different

### Histological examination

**Fig. 5:**
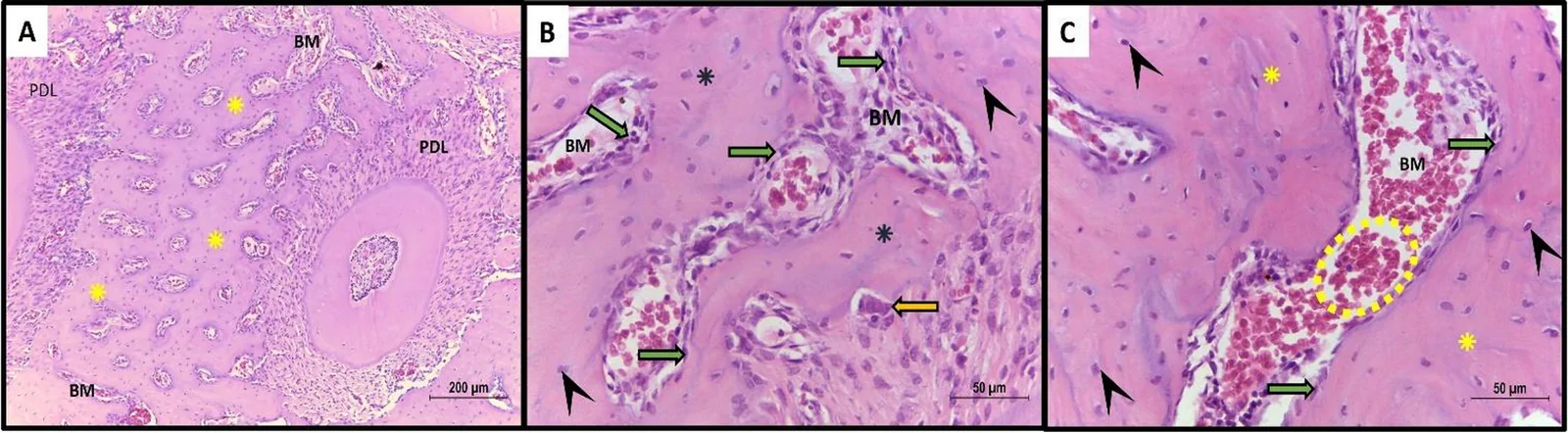
Light microscopic picture of the control group showing, **A** normal bone marrow spaces (BM), bone trabeculae of normal thickness (asterisk) (original magnification X10). **B & C** normal bone marrow spaces (BM) lined with a continuous layer of bone-forming cells (green arrows), and vascular congestion (circles), bone trabeculae of normal thickness (asterisk), multinucleated osteoclast in Howship’s lacuna (yellow arrow), and normal osteocytes in lacuna (black arrow heads). PDL=Periodontal ligaments. (original magnification X40)

**Fig. 6:**
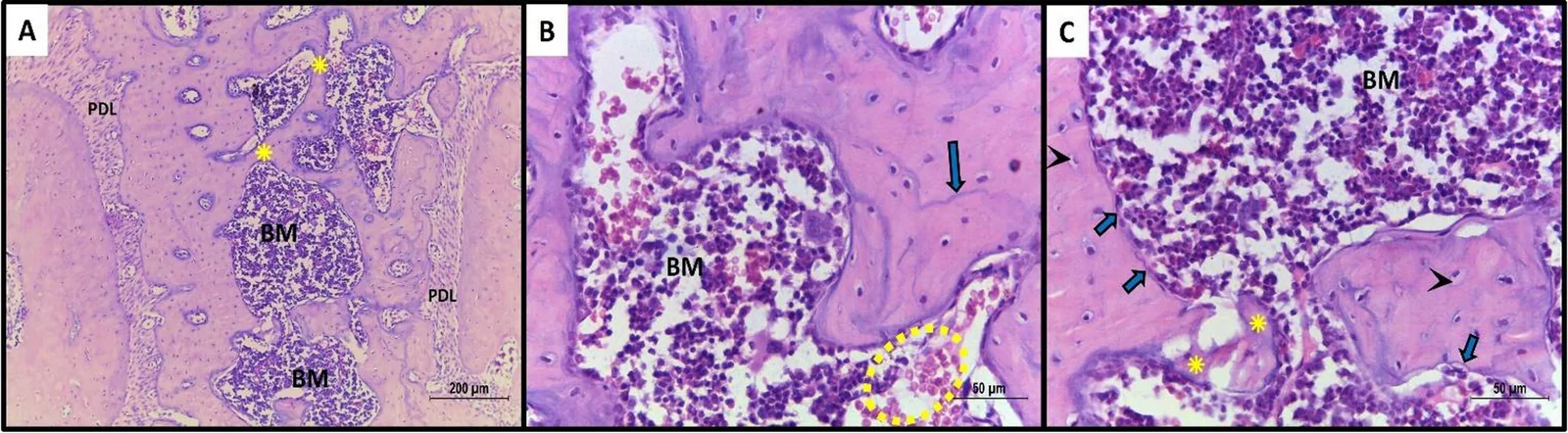
Light microscopic picture of the STZ group. **A** widened bone marrow spaces (BM), thinning bone trabeculae (asterisks)(original magnification X10). **B&C** widened bone marrow spaces (BM) showing vascular congestion (circles), reversal lines (blue arrows), widened osteocytic lacuna (black arrowhead), and areas of bone sequestration (asterisks). PDL=Periodontal ligaments. (original magnification X40)

**Fig. 7:**
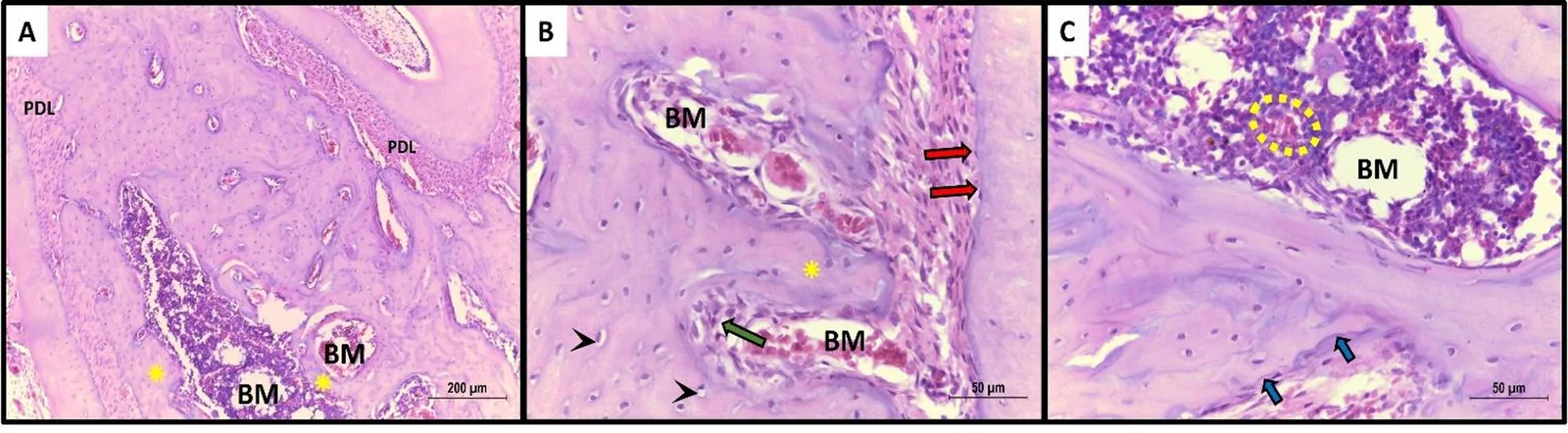
Light microscopic picture of the MET group. **A** areas of widened bone marrow spaces (BM), thinning of bone trabeculae (asterisks)(original magnification X10). **B&C** bone marrow spaces (BM) showing aggregation of bone-forming cells (green arrow), areas of vascular congestion (circles), thinning of bone trabeculae (asterisks), widened osteocytic lacuna (black arrowheads), and reversal lines (blue arrows). PDL=Periodontal ligaments. (original magnification X40)

Histological examination of the control group revealed normal bone marrow spaces enclosed within bone trabeculae of normal thickness, with normal osteocytes present in their lacunae. Bone marrow spaces were lined with a continuous layer of bone-forming cells, and scattered multinucleated osteoclasts residing in Howship’s lacuna were detectable. Areas of vascular congestion within the marrow spaces were noticeable **(**Fig. 5**)**. Histological examination of the STZ group revealed thin, irregular bone trabeculae enclosing widened bone marrow spaces showing vascular congestion. Reversal lines could be observed. Bone trabeculae showed widened osteocytic lacuna and areas of bone sequestration **(**Fig. 6**)**. A similar histological picture was observed in the MET group, with a slight improvement in the widening of bone marrow spaces, along with evidence of active bone remodeling. Multiple reversal lines were evident **(**Fig. 7**)**.

### Histomorphometric analysis

The highest bone area percentage was observed in control group with statistically significant difference between groups (*p* < 0.001, effect size = 0.834). Pairwise comparison showed a statistically significant decrease in bone area percentage in the STZ and MET groups compared to the control group (*p* < 0.001) and (*p* = 0.012), respectively. A significant increase in bone area percentage was detected in the MET group compared to the STZ group (*p* < 0.001).

The highest osteoblast area/bone area was observed in control group with statistically significant difference between groups (*p* < 0.001, effect size = 0.969). Pairwise comparison showed a statistically significant decrease in osteoblast area/bone area in the STZ and MET groups compared to the control group (*p* < 0.001). A significant increase in osteoblast area/bone area was detected in the MET group as compared to the STZ group (*p* < 0.001).

The highest resorbing area/bone area was observed in MET group followed by STZ group with statistically significant difference between groups (*p* < 0.001, effect size = 0.783). Pairwise comparison revealed a significantly higher resorbing area/bone area in both the MET and STZ groups compared to the control group (*p* < 0.001), while the difference between the MET and STZ groups was insignificant (Table 4).

**Table 4:** Descriptive statistics and comparison between groups for morphometric analysis (One-way ANOVA test and Tukey’s post hoc test)

Parameter	Group	Mean ± SD	Std. Error	95% Confidence Interval for Mean	*P* value	Effect size
Bone area percentage	Control	77.30 ± 7.30 ^A^	2.98	(72.00, 82.60)	*P* < 0.001*	0.834
	STZ	47.09 ± 6.19 ^C^	2.53	(41.79, 52.38)		
	MET	65.6 ± 4.42 ^B^	1.80	(60.32, 70.92)		
Osteoblast area/bone area	Control	0.797 ± 0.036 ^A^	0.0145	(0.7529, 0.8407)	*P* < 0.001*	0.969
	STZ	0.193 ± 0.039 ^C^	0.0158	(0.1492, 0.2370)		
	MET	0.339 ± 0.069 ^B^	0.0285	(0.2952, 0.3830)		
Resorbing area/bone area	Control	0.11 ± 0.013 ^B^	0.0052	(0.09, 0.120)	*P* < 0.001*	0.783
	STZ	0.49 ± 0.191^A^	0.078	(0.3874, 0.5882)		
	MET	0.57 ± 0.057 ^A^	0.023	(0.4653, 0.6661)		

Significance level *P* < 0.05, *significant

Means with different superscript letters are significantly different

### Gene expression analysis RunX2, osteocalcin and Col1A1

A decrease in gene expression of osteoblastic markers, including RunX2, Osteocalcin, and Col1A1, was recorded in both the STZ and MET groups relative to the control group. A significant increase in gene expression of osteoblastic markers RunX2, Osteocalcin, and Col1A1 was detected in the MET group as compared to the STZ group (*p* < 0.001)(effect size = 0.979, 0.989 and 0.812 respectively) (Table 5).

**Table 5:** Descriptive statistics and comparison between groups for RunX2, Osteocalcin, and Col1A1 (Student’s T test)

Parameter	Group	Mean ± SD	Std. Error	95% Confidence Interval for Mean	*P* value	Effect size
RunX2	STZ	0.38 ± 0.026	0.0105	(0.3551, 0.4092)	*P* < 0.001*	0.979
	MET	0.798 ± 0.039	0.016	(0.7572, 0.8388)		
Osteocalcin	STZ	0.517 ± 0.017	0.006	(0.4988, 0.5345)	*P* < 0.001*	0.989
	MET	0.81 ± 0.017	0.0061	(0.7876, 0.8234)		
Collagen Type I	STZ	0.247 ± 0.045	0.0183	(0.2128, 0.2813)	*P* < 0.001*	0.812
	MET	0.430 ± 0.051	0.0209	(0.3762, 0.4838)		

Significance level *P* < 0.05, *significant

###  Radiographic assessment

#### Results of the subjective evaluation of the marrow spaces

**Fig. 8:**
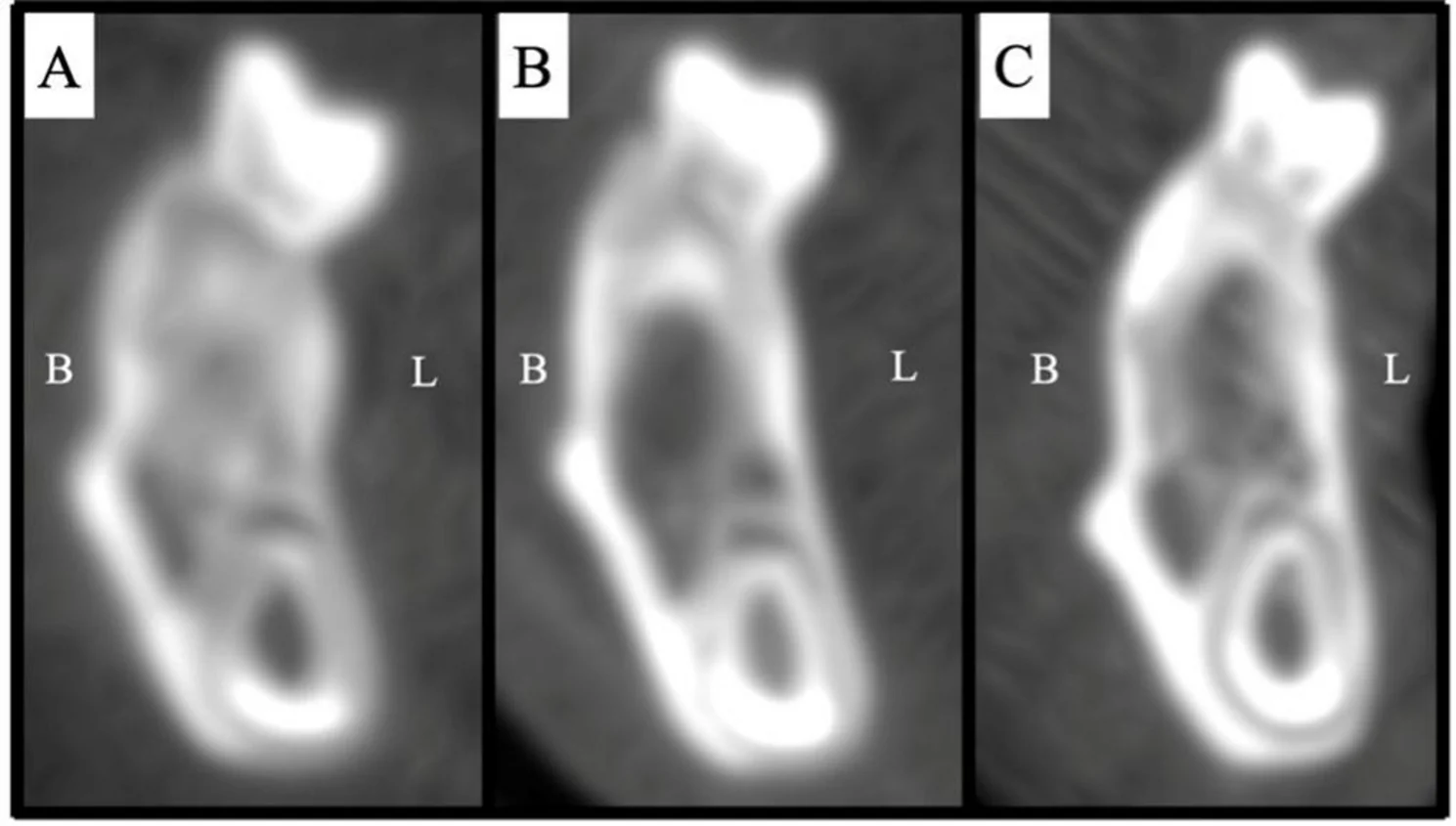
Corrected coronal CBCT images for the three groups: **A** control group with the narrowest marrow spaces, **B** STZ group with the widest marrow spaces & **C** MET group with intermediate marrow spaces

Bone marrow spaces were evaluated for each group. The results showed that the widest marrow spaces were observed in the STZ group, followed by the MET group, while the narrowest marrow spaces were detected in the control group (Fig. 8).

#### Results of measurements of cortical thickness

**Fig. 9:**
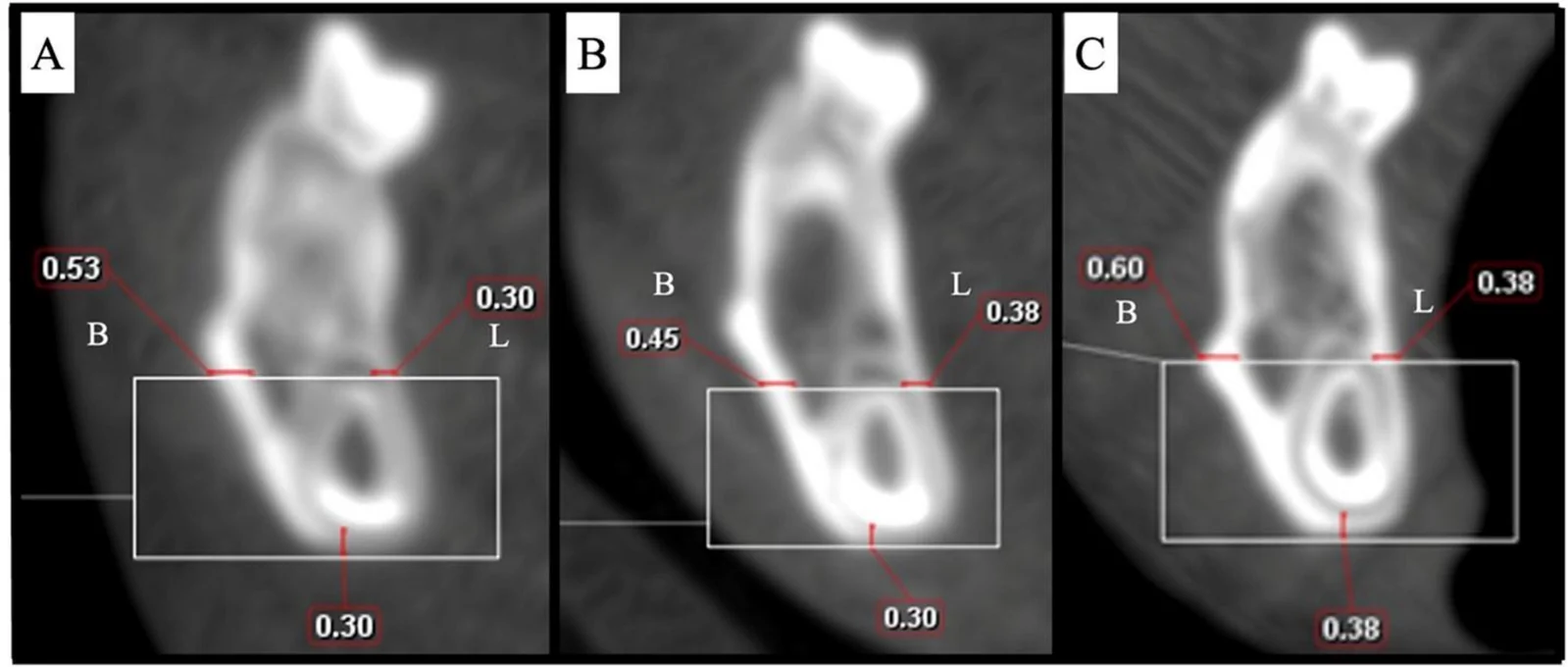
Corrected coronal CBCT images showing the measurements of the cortical bone thickness of the buccal, lingual, and the inferior border of the mandible **A** control group, **B** STZ group & **C** MET group

The results of the cortical thickness measurements for the buccal, lingual, and inferior border of the mandible showed no noticeable differences between the three groups. (Fig. 9).

#### Results of the estimated average gray value assessment

**Fig. 10:**
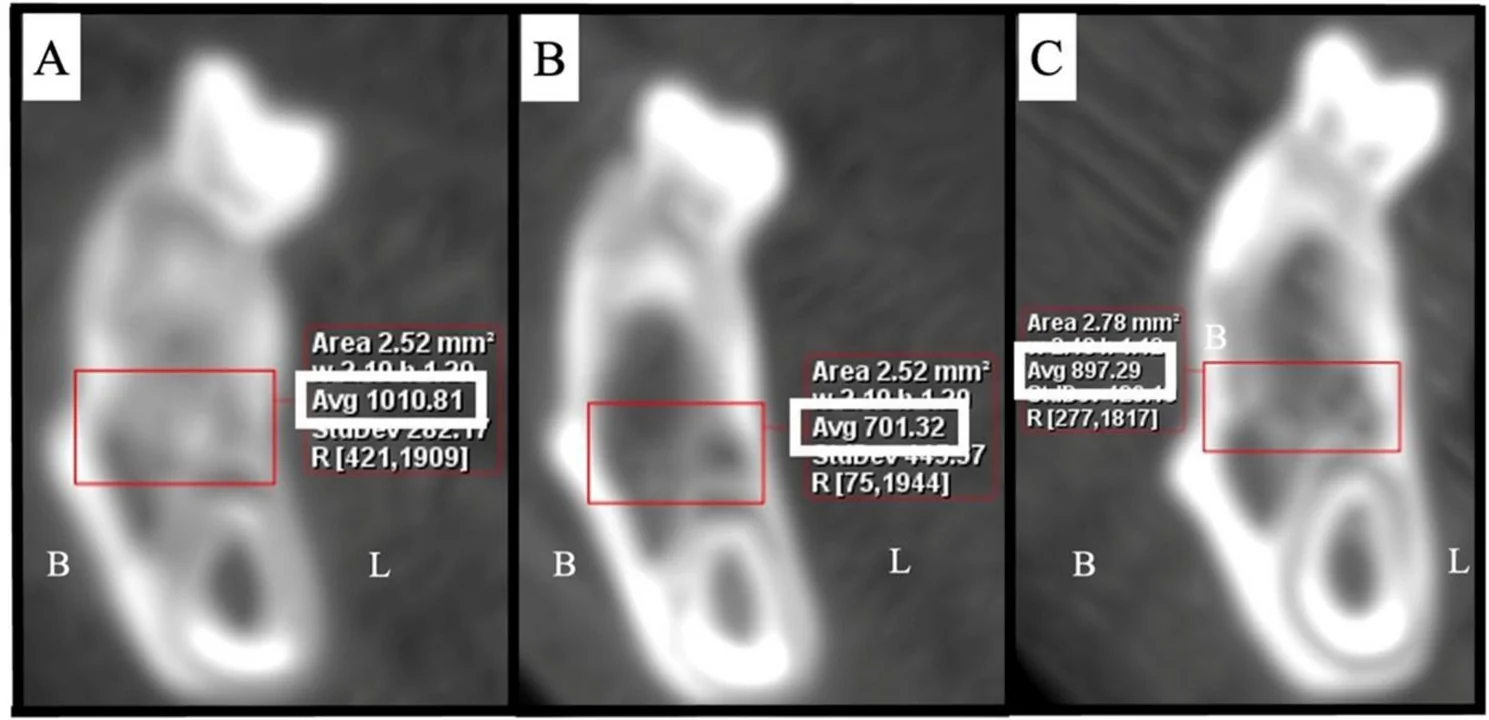
Corrected coronal CBCT images showing a drawn box tool to assess the average estimated gray value; **A** control group, **B** STZ group & **C** MET group

The average estimated gray value for the STZ group was (700–750), for the MET group was (850–900), and for the control group was (1000–1300) (Fig. 10).

## Discussion

Bone remodeling is an essentially continuous process that occurs in the alveolar bone to facilitate tooth eruption and distribute the occlusal forces. One of the significant complications of STZ-induced T2DM is impaired bone metabolism [48]. Furthermore, implant failure is often reported to be associated with T2DM due to poor osseointegration [48].

MET is the first-line treatment for T2DM. Beyond facilitating insulin’s glucose-lowering effects, it was shown to be associated with decreased bone fracture rates and increased osteoblast differentiation in diabetic patients [49]. It has also been demonstrated that MET reduces the inflammatory reaction and bone resorption in models of induced periodontitis in diabetic rats [50].

The purpose of the ongoing study was to assess the short-term effect of MET on alveolar bone integrity and osteoblastic gene expression in a rat model of STZ-induced T2DM. The current study duration was set to be 4 weeks after T2DM induction to evaluate the impact of MET treatment at the early stage of the disease. It was stated that the adverse effects of T2DM on bone were detected as early as 24 days [51] to 5 weeks [52]. Meanwhile, MET treatment of T2DM showed a superior effect on bone reconstruction after 2 weeks [53] to 30 days [50] of administration. These observations suggest that a one-month duration provides a suitable timeframe for identifying early bone metabolic shifts, serving as a baseline to evaluate the potential of MET to initiate metabolic recovery.

The experimental T2DM model was conducted according to the methodology previously described [29]. They concluded that rats fed a 10% fructose solution for 2 weeks, then injected with 40 mg/kg body weight STZ, is an alternative model for T2DM due to its rapid induction time, which effectively induces the pathogenesis of T2DM, IR, and partial pancreatic β-cell dysfunction.

The dose of MET used in the current study was chosen according to previously published studies, which indicated that this dose showed favorable results concerning the repair of damaged tissues and reductions in bone loss over a duration ranging from 2 weeks to one month [54–56]. As other doses may cause different effects, it is recommended that this be investigated in future studies.

The analysis of blood glucose levels and body weight revealed a significant increase in blood glucose levels and a decrease in body weight in the STZ (diabetic) group compared to the control group. Meanwhile, a significant reduction in blood sugar levels and an increase in body weight were observed in the MET group compared to the STZ group. These results are consistent with previous work confirming successful induction of T2DM in experimental animals and the therapeutic effect of MET on glycemic control [29, 30, 57, 58]. This pattern reflects partial β-cell dysfunction characteristic of this T2DM model, alongside diabetes-associated catabolic weight loss, both of which are mitigated by MET’s glucose-lowering action.

Histological, histomorphometric and gene expression analysis results within the current study, revealed a significant improvement in MET treated group as compared to STZ group. These findings are in alignment with previous work that concludes the effect of MET on ameliorating alveolar bone loss in diabetic rats histologically [42].

According to previous studies, untreated DM reduced osteoblastic activity, bone turnover, and bone volume in the mandibular alveolar bone [51, 52]. The decline in bone turnover was evidenced by the simultaneous suppression of both bone resorption and formation [59].

In particular, impairment of glucose metabolism in osteoblasts plays a crucial role in the bone anabolic defects associated with T2DM [60]. It was reported that osteoblasts collected from the alveolar bone of T2DM patients exhibited slower growth, lower cell activity, and calcium nodule formation, accompanied by a significant decrease in alkaline phosphatase (ALP), bone gla protein, and Col1A1 concentration compared to those from non-diabetic individuals [61].

When compared to the STZ group, the current results demonstrated that the MET group showed a significant increase in total bone area percent, osteoblast area/bone area, and osteoblastic gene expression. This result was in accordance with previous in vitro studies, which indicated that MET exhibited osteoinductive potential by upregulating the expression of ALP, RunX2, Col1A1, osteocalcin, and osteonectin genes on human mesenchymal stem cells (MSCs) in vitro [62]. Additionally, it was demonstrated that MET stimulated ALP activity, the formation of mineralization nodules, and the differentiation of osteoblast cell lines in a dose-dependent manner [63].

Another animal study concluded that MET administration effectively mitigated the effects of diabetes on orthodontic tooth movement among T2DM rats, restoring, cathepsin K immunostaining, and normalizing tooth movement while diminishing sclerostin expression [55]. Likewise, MET was demonstrated to improve bone regeneration in critical-sized rat alveolar bone defects [62]. The anti-hyperglycemic effect of MET and its ability to reduce the severity of diabetic adverse effects can help explain the positive impact MET showed on bone-forming cells within the current study.

The primary mechanism of action by which MET promotes osteoblast differentiation and trabecular bone formation was attributed to its ability to increase the activation and phosphorylation of the 5′ adenosine monophosphate (AMP)-activated protein kinase (AMPK) signaling pathway in osteoblast cells [64]. AMPK activation promotes osteoblast differentiation, thereby enhancing the expression of key bone markers, such as Runx2 and osteocalcin [65].

Moreover, a preclinical study has shown that MET can enhance the integration of implants and stimulate the osteogenic differentiation of alveolar bone MSCs by activation of the AMPK/BMP/Smad signaling pathway in a T2DM rat model [48]. However, our study indicates that despite considerable improvement, MET treatment did not effectively normalize bone parameters to levels observed in non-diabetic controls, suggesting the existence of limits to its therapeutic potential, which may reflect irreversible diabetic damage or insufficient duration of intervention. These findings contrast with studies suggesting complete normalization in specific parameters, likely due to differences in the severity of the diabetic model, treatment protocols, or assessment timelines [50, 53]. Sun et al. (2022) have associated metformin-induced upregulation of osteogenic markers with the activation of AMPK pathways, including improved macrophage signaling and inhibition of RANKL [48]. However, the present study did not directly assess AMPK signaling; therefore, the upregulation of Runx2, osteocalcin, and Col1A1 observed here cannot be attributed to AMPK activation based on the current data.

The continued rise in resorbing area/bone area in the MET group in the current study highlights a potential limitation of this monotherapy under diabetic conditions. While previous literature suggests that MET primarily works through osteoblast activation via AMPK-RunX2 pathways, our finding of persistently elevated resorbing area leads us to hypothesize that osteoclast dysregulation may persist independently of this pathway and not be fully addressed by MET alone. As osteoclast-specific molecular markers and direct AMPK-RunX2 signaling were not directly measured in this study, suppression of bone resorption cannot be definitively concluded from our data [66]. Instead, these findings build upon existing knowledge by demonstrating that diabetes-related bone disease has multiple cellular targets that exceed the ability of MET monotherapy to address fully, in comparison with studies highlighting the complete restoration of bone remodeling via AMPK activation exclusively [67, 68]. This osteoblast–osteoclast interaction warrants further investigation.

Regarding radiographic results, the CBCT parameter that estimates the difference in attenuation of X-rays by tissues is referred to as the gray scale or gray value. The gray value is the numerical representation assigned to each voxel (three-dimensional picture element) that corresponds to the amount of attenuation of the x-ray beam by each tissue in a specific area of the scanned object [69]. It has been reported in the literature that gray value can estimate bone density, but the absolute bone density should be calibrated [47, 69–72]. Extensive literature confirms that CBCT gray values are highly susceptible to scanning geometry, scatter radiation, and beam hardening. Furthermore, their mathematical distribution varies significantly depending on acquisition parameters such as peak kilovoltage, milliamperage, and field-of-view dimensions, as well as the selected reconstruction algorithms. Furthermore, the technical constraints governing small-animal CBCT resolution must be acknowledged. Due to voxel-size limitations and inherent scatter artifacts common in low-contrast tissue scanning, the radiographic images exhibited lower baseline contrast than micro-CT. While these resolution thresholds were sufficient for broad macroscopic and linear evaluations, the blurred borders pose an inherent limitation for fine, objective tracking of micro-alveolar parameters [71, 73, 74]. To guarantee data validity despite this constraint, we implemented an optimized, high-resolution cone-beam computed tomography (CBCT) protocol with a minimized field of view (FOV). This setup yields sub-millimeter isotropic voxels, a resolution scale that has been documented in the literature as reliable and sufficient for assessing macroscopic, linear, and structural cross-sectional bone dimensions in rats. Bastami et al. (2018) reported that gray value can be used as an indirect predictor for assessing new bone formation or resorption after bone reconstruction in rabbits with grafted bone [70]. Additionally, Sghaireen et al. (2020) reported that CBCT gray value can predict the presence of osteoporosis more accurately than DEXA scan measurements [75].

By assessing the volume gray value, the current study was conducted following Cassetta et al. 2014 [47], who measured the gray value of both cancellous and cortical bone. It was found that the estimated volume gray value showed higher results in the MET group than in the STZ group, resulting from the greater bone filling of the marrow spaces and cortical bone in the MET group. Additionally, comparing the groups regarding the bone marrow spaces and the bone filling these spaces, the results aligned with the histological findings. The MET group showed a significant increase in total bone area percent than the STZ group. The lack of differences in cortical bone thickness among groups may be attributed to the treatment duration which may have been insufficient to detect meaningful changes in cortical bone. Cortical bone, being less metabolically active than trabecular bone, likely requires longer intervention periods to manifest measurable structural changes. In contrast, trabecular parameters (e.g., marrow space filling, gray values) showed earlier responsiveness to MET.

A study was reported by Shokri et al., 2023 [76] who assessed the effect of different wavelengths of photobiomodulation therapy on the bone mineral density in osteoporotic rats using the measurement of the estimated AGV of CBCT scans of the osteoporotic rats. They found a significant difference between the control and treated groups in AGV. The treated groups showed a lower AGV than the control group, indicating reduced beam attenuation in the treated groups due to increased bone marrow filling.

Moreover, Anbiaee et al. (2022) [46] found that there was a strong correlation between the gray values measured by CBCT and bone volume on the maxillary jaws of sheep. While, Suttapreyasri et al. [77] found a strong association between the cortical bone thickness and bone volume, with no statistically significant association between BV/TV and GVs. In contrast to these results, Corpas Ldos et al. [78] found there was no correlation between gray values and histomorphometric analysis of the peri-implant bone. But they did not consider the metal artifact around the implant.

Within the limitations of the current study, it focuses only on baseline bone conditions, lacks monitoring of insulin resistance measurements in diabetic rats, investigates only one duration and a single MET dose, and lacks osteoclast markers. Specifically, Insulin resistance was inferred from the validated fructose-STZ model rather than directly quantified through HOMA-IR or fasting insulin measurements, and only male rats were studied, despite documented sex-specific differences in diabetic bone disease. The absence of osteoclast-specific markers (RANKL, OPG, TRAP, CTSK) means that the persistent elevation in resorbing area observed in the MET group—despite improvements in osteoblastic parameters—supports, but cannot directly demonstrate, the proposed model in which MET acts predominantly on osteoblastic pathways while leaving osteoclast dysregulation largely unaddressed. Future work should address surgical or implant-related scenarios, incorporating dose-response and time-course designs, direct measures of insulin sensitivity, both sexes, and a complete panel of osteoblastic and osteoclastic markers will be required to test this mechanistic hypothesis and to determine whether full normalization of alveolar bone parameters is achievable with MET alone or only with adjunctive therapy. Additionally, as no phantom calibration or validated density-conversion method was used, AGV values should be interpreted as relative radiographic attenuation estimates rather than as absolute measures of bone mineral density.

## Conclusion

The findings of the present study demonstrate that 30 days of MET administration significantly improved alveolar histomorphometric and molecular parameters in T2DM rats, as evidenced by increased total bone area percentage and upregulated osteoblastic gene expression (Runx2, osteocalcin, and Col1A1) compared with untreated diabetic rats. Histological observations further supported these improvements, showing enhanced bone remodeling, while radiographic assessments indicated increased AGV, reflecting greater X-ray attenuation, and narrower marrow spaces. Despite these benefits, MET did not fully restore bone parameters to the levels observed in non-diabetic controls. Overall, these findings provide preclinical evidence that MET offers partial protection against short-term diabetes-associated alveolar bone loss in this rat model. Further translational research is needed to determine whether longer treatment durations, alternative doses, or adjunctive strategies can achieve more complete bone restoration before extrapolating these findings to clinical practice. Given that osteoclasts influence bone formation by producing factors that stimulate osteoblasts, future studies should investigate how MET affects the molecular mechanisms linking these two cell types.

## Data Availability

All data will be available from the corresponding author upon reasonable request.
